# Antiviral therapy for HCV in hemophilia A patients with HIV-1 co-infection

**DOI:** 10.1097/MD.0000000000016524

**Published:** 2019-07-26

**Authors:** Hong Xiao, Jun Chen, Jiangrong Wang, Juhua Li, Feia Yang, Hongzhou Lu

**Affiliations:** Department of Infectious Diseases, Shanghai Public Health Clinical Center, Fudan University Shanghai, PR China.

**Keywords:** co-infection, direct-acting antiviral, HCV, hemophilia, HIV

## Abstract

Anti-hepatitis C virus (HCV) treatment for human immunodeficiency virus (HIV)/HCV co-positive patients with hemophilia A presents numerous problems in terms of safety and effectiveness. The emergence of direct-acting antiviral (DAA) regimens has led to tremendous changes in the management of HIV/HCV co-infection over the past few years, but the application of DAA in patients with hemophilia complicated with HIV/HCV co-infection has rarely been reported.

We retrospectively analyzed the clinical course and outcome of hemophilia A patients with HIV/HCV co-infection receiving DAA with a focus on the virological response, changes in cluster of differentiation 4 lymphocyte (CD4) count, side effects, and impact on bleeding before and after DAA therapy.

A total of 12 hemophilia A patients with HIV/HCV co-infection were included, 9 of which were severe. All the patients were in stable states with CD4 counts >200/mm^3^ and plasma HIV ribonucleic acid (RNA) suppressed (<40 IU/mL) while taking the antiretroviral regimen. Majority of the patients (n = 9, 75.0%) were infected with HCV genotype (GT) 1b, while 2 and 1 was infected with HCV GT 2i and HCV GT 3, respectively.

After 12 weeks of DAA treatment, 11 patients (91.7%) obtained sustained virologic response within 24 weeks of discontinuation of treatment (SVR24), except 1 patient who was treated with sofosbuvir (SOF) + pegylated interferon + ribavirin (PR), which was then switched to daclatasvir (DCV) + asunaprevir (ASV) for 12 weeks; this patient then achieved SVR24. During DAA treatment, HIV RNA in all the patients was constantly suppressed, while CD4 counts showed no obvious change.

The most common treatment-emergent adverse events were weakness and loss of appetite (generally mild). There was no evidence of an increased tendency of bleeding, and changes in response to replacement.

DAA therapy offered a safe and well-tolerated management strategy for HIV/HCV co-infected patients with hemophilia A. An awareness of the potential drug-drug interactions (DDI) between DAA and combination antiretroviral therapy (cART) by clinicians is important for optimal management of co-infected patients.

## 1 Introduction

The global burden of disease attributed to HIV and HCV infection is substantial. Some hemophiliacs are infected with HIV and or HCV due to infusion of contaminated blood products.^[1,2]^ HCV and HIV can impact each other's natural processes,^[3,4]^ with accelerated liver disease progression and increased acquired immune deficiency syndrome (AIDS)-related morbidity, even in those receiving cART.^[5]^ Although the number of HIV-related deaths is falling, that of HCV-related liver disease deaths is rising. Thus, management of HCV infection should be prioritized in patients with HIV coinfection.^[6]^

Interferon-based treatment in patients with HIV/HCV co-infection has low rate of sustained virological response (SVR) and significant toxicities, limiting the application of the drug. Although SVR reduces both liver- and non-liver-related complications and mortality, therapy with PR resulted in SVR in less than 30% of HIV-positive individuals with HCV GT1.^[7,8]^ In 2011, the first generation of DAA HCV protease inhibitors (boceprevir and telaprevir) were introduced. Combined with PR for HCV GT1, infection in patients with HIV in pivotal clinical trials have been reported,^[9,10]^ the SVR rates increased to 63% and 74%, respectively. Subsequently, new drugs were developed, including non-structural protein 5B (NS5B) RNA polymerase inhibitor and non-structural protein 5A protease inhibitor and so on. The availability of DAA for HCV offers considerable option in the management of HCV. It has a high efficacy, is well-tolerated, and a shorter treatment duration.^[11,12]^ From the results of large-scale clinical studies, the efficacy of DAA in the treatment of HIV/HCV co-infected patients was consistently comparable with that of HCV infection alone; the current guidelines no longer distinguish between these 2 types of patients.

However, due to the existence of similar/common metabolic pathways, attention must be paid to the DDI between DAA and cART regimens. On the other hand, the antiviral treatment of HIV/HCV positive patients with hemophilia A exhibits many problems of safety and effectiveness. For infection with HCV, most of the patients have varying degrees of liver damage, and of bleeding tendency; therefore, in DAA treatment for HCV infection with hemophilia A, side effect of DAA and effect on bleeding disorders must be considered, as well as DDI between DAA and cART.

However, the impact of DAA on hemophilia is not yet very clear.^[13]^ The aim of this analysis was to assess the clinical efficacy and safety of DAA therapy for HCV in hemophilia A patients with HIV co-infection.

## 2 Materials and methods

### 2.1 Patient population and design

This study was conducted in Shanghai Public Health Clinical Center (SHAPHC). Hemophilia A is a genetic deficiency in coagulation factor VIII which causes increased bleeding. The severity of bleeding in hemophilia A is generally correlated with the clotting factor level as follows: mild: >5%, moderate: 1% to 5%, severe: <1%.^[14]^ Some hemophilia A patients, infected with HIV/HCV through contaminated coagulation factor from 1984 to 1985, initiated cART between the years of 1994 and 1996, and achieved undetectable HIV RNA thereafter. From May 2014 to October 2017, 12 hemophilia A individuals with HIV/HCV co-infection received DAA treatment for 12 weeks and were followed up for 24 weeks after treatment. Patients with positive hepatitis B surface antigen and/or hepatitis B core antibodies positive were excluded from this therapy. We retrospectively analyzed the use of DAA therapy in all patients after extracting their clinical data from the original records and reviewing their medical charts and laboratory findings before and during treatment with DAA therapy. Importantly, the virological response, CD4 counts changed from the baseline values, and clinical status were assessed according to the clinical data. The primary endpoint of DAA treatment was the SVR24. Safety was assessed by the incidence of drug associated events and effects on bleeding, which were identified by determining the causal relationship between DAA and the adverse event including bleeding. The safety data were collected for all patients from initiation of DAA. The clinicians were asked to assess the potential causal relationship between DAA and the adverse event including bleeding and to record whether it was judged to be drug-associated. Moreover, based on the original medical records, we calculated the depletion of coagulation factor VIII during DAA treatment to assess the hemorrhagic tendency and response to replacement therapy of the patients.

The study was approved by the Ethics Committee of the research institution. All the authors had access to the research data and agreed to keep the data confidential. The final manuscript was reviewed and approved by all the authors.

### 2.2 CD4 count, HCV RNA measurement, and HCV GT detection

EDTA anticoagulated blood samples for CD4 count was by flow cytometry (BD Company, USA, CYTOMICS-FC500) at SHAPHC. In this study, we defined the baseline CD4 count as the last test performed within one month before DAA was initiated.

HCV RNA quantitative detection were performed at baseline, weeks 2, 8, and 12 during treatment, and at weeks 12 and 24 after the treatment. HCV RNA was measured with the COBAS TaqMan HCV test with a lower limit of detection of 40 IU/ml. Samples shown to be positive for the presence of HCV by reverse transcriptase- polymerase chain reaction (RT-PCR) testing were analyzed further to identify the HCV genotype (GT) using an immune line probe assay (INNO-LiPA, Bayer Diagnostics, US).

### 2.3 Liver stiffness measurement

Fibro Scan (Echosens, France) was performed by a skillful operator to assess the liver stiffness. Ten liver stiffness measurements were recorded, and the median value calculated by the equipment's statistical analysis system was taken as the final score. The following liver stiffness cutoff values were used for staging: F0 / F1, <7.1 kPa; F1 / F2, ≥7.1 kPa; F2, ≥8.7 kPa; F3, ≥9.5 kPa; F3 / F4, ≥12.5 kPa; F4, ≥14.5 kPa.

### 2.4 Ethics statement

The study was approved by the Ethics Committee of SHAPHC. As a retrospective study, the requirement for informed consents was waived.

### 2.5 Statistical methods

This study was not designed as a prospective study to assess treatment; hence, the sample size has not been calculated. This was a retrospective study in which all patients received rigorous treatment.

## 3 Results

### 3.1 Clinical characteristics of study subjects

Twelve hemophilia A individuals with HIV/HCV co-infection were included in this study. Each patient completed the course of treatment and had complete data at follow up. Demographic characteristics are presented in Table 1. All the participants were male and had well-controlled HIV-infection with HIV load <40 IU/ml, CD4 counts >200 cells/mm^3^, and no AIDS-related disease. HCV RNA was detected in all the twelve patients. Among them, 7 patients previously had treatment failure with PR; 2 could not receive interferon therapy (1 for thyroid dysfunction, another had heart disease); and the remaining 3 were initially treated patients. Fibrosis stage was graded in all patients by Fibro Scan within 3 months of enrollment, 4 patients were diagnosed with decompensated cirrhosis.

**Table 1:**
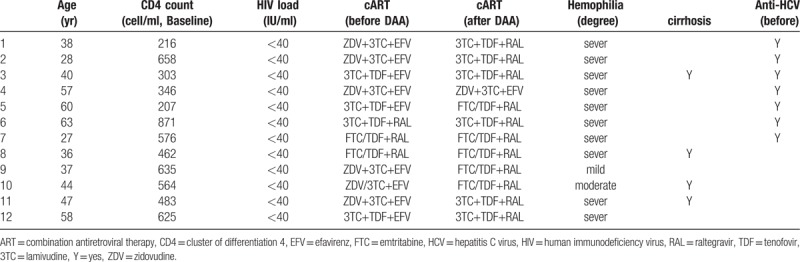
Demographic and clinical characteristics at baseline.

Majority of the patients (9, 75.0%) were infected with HCV GT1b, while the others: 2 infected with GT2i and 1 with GT3. Of the 9 patients with GT1b, 6 received DCV + SOF, 2 received SOF / VEL and 1 received SOF + PR. Of the 2 patients with GT2i, 1 received DCV + SOF, and the other received SOF + ribavirin (RBV). The patient with GT3 received DCV + SOF for 12 weeks (Table 2). The information on antiretroviral therapy before and after DAA treatment is described in Table 1. Most patients’ cART regimens included efavirenz (EFV), which had significant DDI with DAA regimens. The most common cART regimens were zidovudine (ZDV) + lamivudine (3TC) +EFV. We evaluated for the potential DDI between selected DAA and cART received by each individual based on the latest literature, and adjusted the cART according to each patient's DAA program referencing guidelines.

**Table 2:**
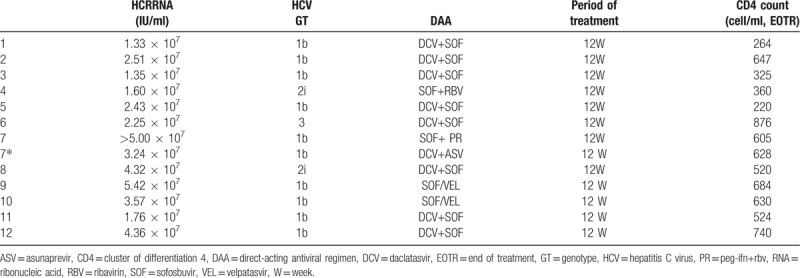
The patient's HCV load, genotype and DAA regimens.

### 3.2 The efficacy of DAA

**Figure 1:**
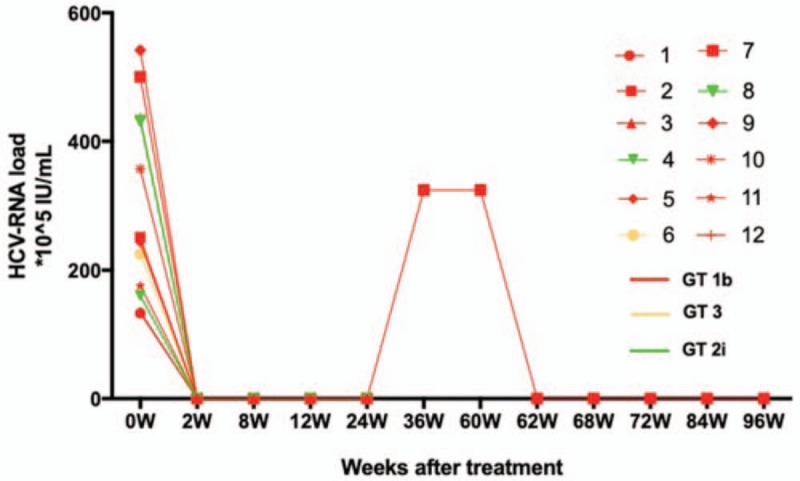
The process of anti-HCV treatment with DAA. The 12 patients completed the 12 weeks of treatment; the HCV RNA became undetectable by week 2. In the end, 11 patients achieved SVR24, anti HCV therapy failed in 1 patient infected with GT1b HCV who was treated with SOF + PR. HCV breakthrough occurred at 24 weeks after the end of treatment, the HCV RNA load rose to 3.24 × 10^7^ IU/ml. 6 months later, the patient received DCV + ASV for 12 weeks; HCV RNA became undetectable within 2 weeks of treatment, and achieved SVR24. ASV = asunaprevir, DCV = daclatasvir, GT = genotype, HCV = hepatitis C virus, RNA = ribonucleic acid, SVR24 = sustained virological response within 24 weeks of discontinuation of treatment, W = week.

All patients completed the 12 weeks of treatment; the HCV RNA became undetectable by week 2, and sustained virological response within 12 weeks of discontinuation of treatment (SVR12) was achieved in all patients. Eleven patients achieved SVR24. Anti HCV therapy failed in 1 patient infected with GT1b HCV, and he was treated with SOF + PR. HCV breakthrough occurred at 24 weeks after the treatment completion. The HCV RNA load rose to 3.24 × 10^7^ IU/ml and 6 months later, the patient received DCV + ASV for 12 weeks; thus, HCV RNA became suppressed to undetectable levels within 2 weeks of treatment, and SVR24 was achieved (Fig. 1). During treatment, HIV RNA remained constantly undetectable and the CD4 counts stabilized at the baseline levels in all patients (Tables 1 and 2).

### 3.3 Side effects of DAA treatment

Overall, only weakness and loss of appetite were noted in 2 patients on SOF/ VEL for HCV GT1, but none was reported as serious or leading to discontinuation. No worsening of blood cell count, lipodystrophy, or liver function was observed (data not shown). The DAA regimen was suitable for co-administration in all patients.

Severe hemophilia A Patients were regularly infused with coagulation factor VIII (30 IU/kg, 3–4 times a week) for secondary bleeding prevention. There was no significant change in the monthly usage by each patient (data not shown). In addition, there was no increased bleeding frequency or any change in the efficacy of the replacement therapy with clotting factors in all the patients.

## 4 Discussion

HCV infection is the main cause of mortality in hemophilia patients.^[15,16]^ Even in patients receiving cART, an increasing proportion of deaths were related to liver disease among patients with hemophilia and HCV co-infection.^[17,18]^ Thus, these has significant implications for the choice of anti-HCV treatment.

The availability of highly effective, well-tolerated DAA regimens for HCV should diminish barriers to therapy in HIV/HCV coinfection. However, DAA demonstrates interaction with cytochrome P450 enzymes or transporters, which have similar metabolic pathways with most anti-HIV drugs; therefore, combined medication may increase the blood concentration of drugs to cause serious adverse reactions or reduction in blood concentration, thus affecting the efficacy. An awareness of the potential DDI between DAA and cART drugs by clinicians is important for optimal management of co-infected patients.^[19]^

In many districts with limitations or restrictions of DAA or cART, in order to reduce the occurrence of DDI, antiretroviral switches may be performed to allow for the co-administration with specific DAA.^[20]^ Based upon the current guidelines^[21]^ and literatures,^[22]^ integrase inhibitors and nucleoside reverse transcriptase inhibitors are less likely to develop DDI with DAA. Unlike protease inhibitor (PI) and non-nucleoside reverse transcriptase inhibitors (NNRTI), such as 3TC, emtricitabine (FTC), abacavir, and rategravir (RAL); dolutegravir can be co-administrated safely with DAAs recommended in the current guidelines. EFV as a moderate P-glycoproten inducers or moderate cytochrome enzyme inducers may decrease DCV and VEL plasma concentration leading to reduced therapeutic effect, Therefore, the co-administration of EFV containing regimens with SOF / VEL is not recommended. When it is combined with DCV, the daily dose of DCV should be increased to 90 mg. Thus, for patients with co-infection who are started on treatment with NNRTI or PI-based cART, it is appropriate to replace these with an integrase inhibitor-based cART. If the patient has more comorbidities, taking multiple drugs, we must be cautious when using DAA. By visiting a website (http://www.hep-druginteraction.org), a quick search can be made for DDI and treatment options between DAA and other combined medications.

In the current study, all the HIV/HCV co-infected hemophilia A patients received DAA therapy and achieved SVR24 regardless of previous history of anti-HCV treatment with PR or for compensated cirrhosis. One patient, who was initially treated with PR, had treatment failure with SOF + PR, but when he was switched to DCV + ASV for 12 weeks, he achieved SVR24. The reason for the treatment failure is believed to be related to the high viral load before treatment, the history of PR treatment, and more importantly, the selected treatment options. The SOF + PR regimen were previously recommended for the treatment of GT1 infections,^[23]^ but real-world data suggest a 70% regimen effectiveness with higher recurrence rate; thus, it is not recommended for patients with GT1 infection.^[24]^

In patients with hemophilia A, DAA therapy was well-tolerated, no patient discontinued with the treatment due to side effects; however, 2 patients consistently had weakness and loss of appetite, as usually seen in non-hemophilia patients. Additionally, no patient had an increased tendency of hemorrhage, or increased consumption of coagulation factor VIII. During the treatment and follow-up, no patients had HIV RNA rebound, and CD4 count did not change significantly.

In the future, with the emergence of new pan-GT DAA, especially the fixed compound preparations, the treatment of HCV is likely to be simpler and safer.

The limitation of this study was the small sample size and relatively few DAA options; this limited the accurate description of the results. A larger sample sized future study may be needed to establish our findings.

Evidence from our study data indicate that DAA provided a safe and effective treatment option for hemophilia A patients with HIV/HCV co-infection as with another report.^[25]^ However, suitable treatment regimen needs to be selected to avoid DDI; and careful monitoring of adverse reactions is necessary.

## Author contributions

**Data curation:** Jiangrong Wang, Juhua Li, Fei Yang.

**Formal analysis:** Hong Xiao, Jun Chen.

**Investigation:** Jun Chen, Jiangrong Wang, Juhua Li, Fei Yang.

**Project administration:** Hongzhou Lu.

**Supervision:** Hongzhou Lu.

**Writing - original draft:** Hong Xiao.

**Writing - review & editing:** Hong Xiao, Jun Chen, Hongzhou Lu.
